# Pinostrobin, a fingerroot compound, regulates miR-181b-5p and induces acute leukemic cell apoptosis

**DOI:** 10.1038/s41598-023-35193-6

**Published:** 2023-05-19

**Authors:** Chosita Norkaew, Paweena Subkorn, Chawalit Chatupheeraphat, Sittiruk Roytrakul, Dalina Tanyong

**Affiliations:** 1 Department of Clinical Microscopy, Faculty of Medical Technology, Mahidol University, Nakhon Pathom, 73170 Thailand; 2 Center for Research and Innovation, Faculty of Medical Technology, Mahidol University, Nakhon Pathom, 73170 Thailand; 3 Functional Proteomics Technology Laboratory, Functional Ingredients and Food Innovation Research Group, National Center for Genetic Engineering and Biotechnology, National Science and Technology for Development Agency, Pathum Thani, 12120 Thailand

**Keywords:** Cancer, Cell biology

## Abstract

Pinostrobin (PN) is the most abundant flavonoid found in fingerroot. Although the anti-leukemic properties of PN have been reported, its mechanisms are still unclear. MicroRNAs (miRNAs) are small RNA molecules that function in posttranscriptional silencing and are increasingly being used in cancer therapy. The aims of this study were to investigate the effects of PN on proliferation inhibition and induction of apoptosis, as well as the involvement of miRNAs in PN-mediated apoptosis in acute leukemia. The results showed that PN reduced cell viability and induced apoptosis in acute leukemia cells via both intrinsic and extrinsic pathways. A bioinformatics approach and Protein–Protein Interaction (PPI) network analysis revealed that ataxia-telangiectasia mutated kinase (ATM), one of the p53 activators that responds to DNA damage-induced apoptosis, is a crucial target of PN. Four prediction tools were used to predict ATM-regulated miRNAs; miR-181b-5p was the most likely candidate. The reduction in miR-181b-5 after PN treatment was found to trigger ATM, resulting in cellular apoptosis. Therefore, PN could be developed as a drug for acute leukemia; in addition, miR-181b-5p and ATM may be promising therapeutic targets.

## Introduction

Leukemia, a hematologic cancer, is characterized by irregularities in white blood cell production. These dysfunctional leukocytes weaken their ability to fight infection and inhibit the production of healthy blood cells in the bone marrow^1^. According to statistics from GLOBOCAN, leukemia is the 11th most common malignancy in the world, with a high mortality rate. In 2020, there were 311,594 leukemia deaths and 474,519 new cases^2^. Chemotherapeutic drugs are frequently used to treat leukemia. However, they deplete the number of leukemia cells as well as healthy blood cells, which causes anemia and raises the danger of bleeding and infection. Moreover, they can cause nausea, vomiting, hair loss, and a loss of appetite^3^. Due to the various side effects of chemotherapy, studies to increase therapeutic efficacy are becoming more and more interested in finding new possible compounds from natural products. From 1946 to 2019, over 79% of anticancer medications that have been approved come from natural sources^4,5^, including herbs, which represent one of the primary sources of phytochemicals, or biologically active substances. Additionally, herbs are easily accessible, inexpensive, and relatively nontoxic^6^. Numerous phytochemicals exhibit antitumor and antileukemic activities through a variety of mechanisms. While some can suppress proliferation, invasiveness, and angiogenesis of tumors, some can induce cancer cell death by acting on many targets and signaling pathways, such as kinases, cyclins, pro-apoptotic, and tumor suppressor proteins^6,7^. Therefore, the use of phytochemicals has had a significant impact on the discovery of new drugs.

Pinostrobin (5-hydroxy-7-methoxy flavanone: PN) is the most abundant flavonoid found in fingerroot (*Boesenbergia rotunda*), a common food ingredient and traditional herbal medicine in many Asian countries^8^. It displays a wide range of bioactivities, including antioxidant, antibacterial, and antiviral activities^9–11^. Previous research has indicated that PN has anticancer effects by reducing cell growth and promoting cell apoptosis in several cancers, including glioblastoma, breast cancer, ovarian cancer, and leukemia^12–14^.

Apoptosis mechanisms in cancers involve many regulators, including microRNAs (miRNAs). miRNAs are small noncoding RNA molecules with a length of 18–25 nucleotides. Numerous cellular processes, including proliferation, growth, and death, are regulated by miRNAs. miRNAs suppress their target genes by binding to a specific sequence in the 3′ untranslated region (3′UTR) of target mRNAs, leading to translational repression and mRNA cleavage^15^. Depending on their target genes, they can either be oncogenes or tumor suppressor genes. Many studies have demonstrated that several cancer types can alter miRNAs, and these alterations are linked to both the development of cancer and tumor growth^16^. Several phytochemicals have been reported to have anticancer properties by correcting cancer-specific miRNA changes. For example, (6)-gingerol has been reported to increase the expression of miR-27b, a typically downregulated miRNA in acute myeloid leukemia (AML). This (6)-gingerol-corrected miRNA was important for its antiproliferative and apoptotic induction effects^17^. A combined treatment of curcumin, piperine and taurine in hepatocellular carcinoma patients was able to decrease the circulating levels of miR-21, one of the widely studied oncogenic miRNAs^18^. As a result, miRNAs are increasingly being used as biomarkers for cancer diagnosis and prognosis and as therapeutic targets.

A crucial phase in the drug discovery process is determining the targets of novel drugs. Many experiments are required at this step, which is an expensive and time-consuming process. Fortunately, recent computational methods and bioinformatics tools can help address these drug discovery issues^19^. A ligand-based approach is a computational method that uses the similarity principle to predict drug targets. Structurally homologous molecules frequently display similar features by binding to similar protein targets. Therefore, this method predicts targets for the compound of interest by inferring target molecules of bioactive compounds that are comparable to the compound of interest^20,21^. As proteins typically regulate one another, understanding the molecular mechanisms of new drugs is also essential information for drug development. Protein‒protein interaction networks (PPIs) are promising bioinformatic approaches for exploring the underlying mechanism of new drugs since they provide details about the relationship between drug targets and other proteins^22^. Some studies have demonstrated the anti-leukemic activity of PN, but the mechanism has not been addressed. Furthermore, there have been no reports regarding the involvement of miRNA in PN-mediated apoptosis in leukemia.

In this study, we examined the effect of PN on cell viability and apoptosis in acute myeloid and lymphoid leukemia cells, identified the target proteins and molecular mechanism of PN using a ligand-based approach and PPI construction, and studied the involvement of miRNA in PN-mediated apoptosis in acute leukemia.

## Results

### Pinostrobin exhibits cytotoxicity against acute leukemia cell lines

**Figure 1:**
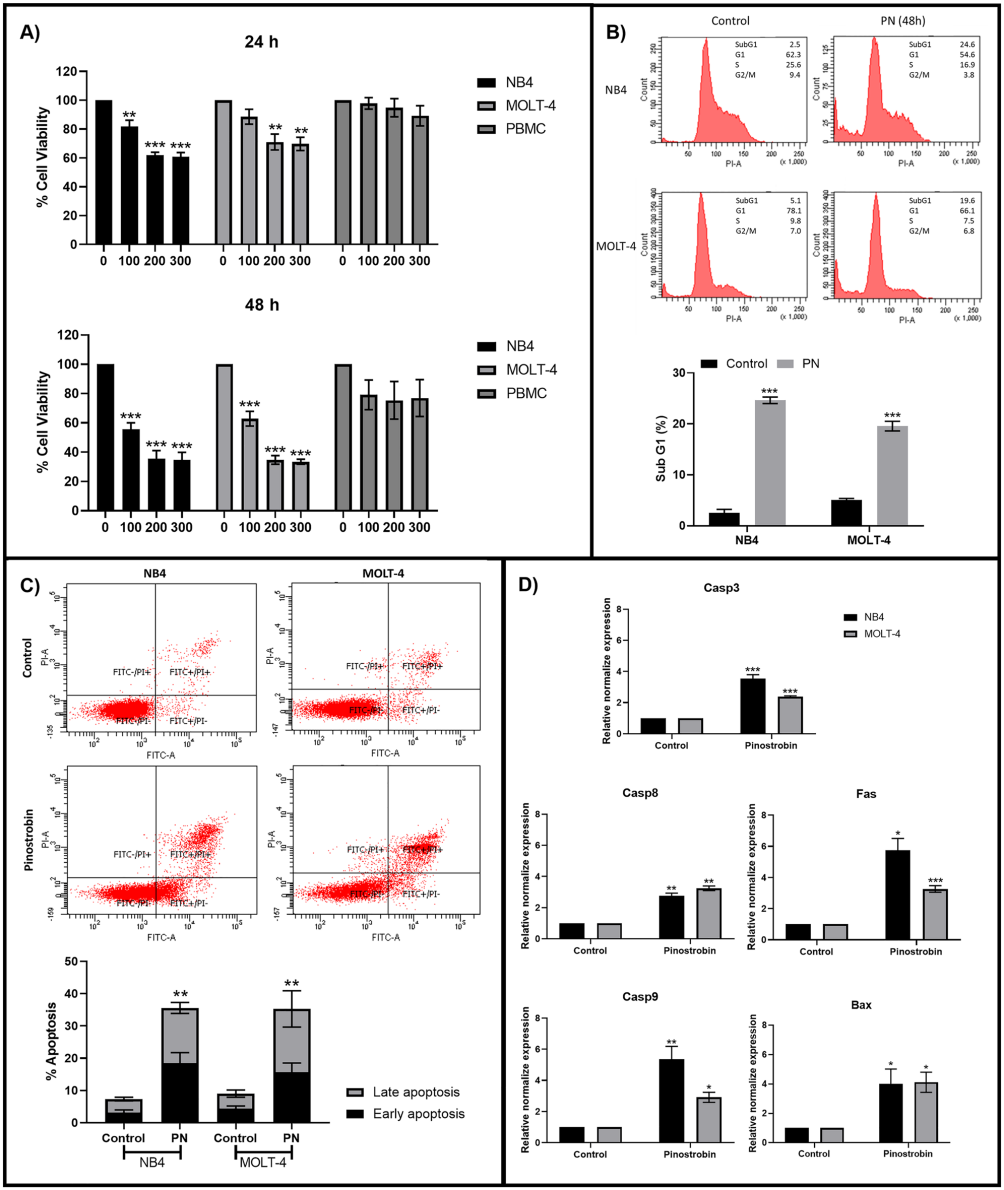
Pinostrobin repressed cell viability and induced apoptosis in acute leukemia cells. (**A**) The viability of NB4, MOLT-4, and PBMC after PN treatment for 24 and 48 h was examined by the MTT assay. (**B**) The cell cycle of NB4 and MOLT-4 was investigated by PI staining. A summary of the percentage of sub-G1 was shown in the bar graph. (**C**) The percentage of apoptosis was determined by cytometric analysis with Annexin V-FITC/PI staining. The quantified results of the apoptosis assay were presented in a bar graph. (**D**) The effect of PN on the mRNA expression of apoptotic genes, including caspase 3, caspase 8, caspase 9, BAX, and Fas, was examined by RT-qPCR. All data was presented as the mean ± SEM of three separate experiments. *P < 0.05, **P < 0.01 and ***P < 0.001 were the statistically significant differences from the control group.

To investigate the cytotoxicity of PN to acute leukemia, the cell viability of NB4 and MOLT-4 leukemia cell lines under PN treatment for 24 and 48 h was observed using the 3-(4,5-dimethylthiazol-2-yl)-2,5-diphenyl-2H-tetrazolium bromide (MTT) assay. As shown in Fig. 1A, PN significantly reduced the cell viability of both leukemia cell lines in a dose- and time-dependent manner. The half-maximal inhibitory concentration (IC50) of PN against NB4 and MOLT-4 at 24 h was 433 ± 84 and 604 ± 157 µM, while the IC50 at 48 h was 132 ± 42 and 142 ± 24 µM, respectively. The viability of peripheral blood mononuclear cells (PBMCs) was unaffected by PN. These findings indicate that PN possesses a cytotoxic effect on NB4 and MOLT-4 leukemia cells without toxicity to healthy PBMCs.

### Pinostrobin induces apoptosis in acute leukemia cells

Propidium iodide (PI) staining was used to examine the cell cycle of PN-treated and control cells, followed by flow cytometry detection. The results, presented in Fig. 1B, demonstrated that PN increased the percentage of sub-G1 phase in both NB4 and MOLT-4 cells by 24.63 ± 0.64% and 19.57 ± 0.95%, respectively. These results indicated that PN could induce cell death with fragmented low-molecular-weight DNA in acute leukemia. DNA endonucleolytic cleavage is one of the common features found in apoptosis, a mechanism of cell death that has long been linked to the treatment of cancer. To confirm whether PN exerts any influence on apoptosis in acute leukemia cells, the percentage of apoptotic cells was measured using Annexin V conjugated with fluorescein isothiocyanate (FITC) and PI staining, followed by flow cytometry detection. After 48 h of incubation with the IC50 of PN, the total percentage of apoptotic cells was increased in NB4 and MOLT-4 cells, with values of 35.57 ± 4.23% and 35.27 ± 3.47%, respectively, as shown in Fig. 1C. According to these findings, PN causes acute leukemia cells to undergo apoptosis, which leads to cell death.

### Alterations in apoptotic genes in acute leukemia cells after pinostrobin treatment

To investigate the mechanism of PN on the induction of apoptosis in NB4 and MOLT-4 cells, the expression of apoptotic genes, including caspase-3, caspase-8, caspase-9, Fas cell surface death receptor (Fas), and Bcl-2-associated X protein (BAX), was examined after 48 h of treatment with PN at the IC50 using reverse transcription-quantitative polymerase chain reaction (RT‒qPCR). The results are shown in Fig. 1D. PN increased the mRNA expression levels of caspase-3, caspase-8, caspase-9, Fas, and BAX in both cell lines. These results indicated that PN-induced apoptosis in acute leukemia cells involves caspase-dependent mechanisms. Notably, PN activated both intrinsic pathways via BAX and caspase-9 and extrinsic apoptotic pathways via Fas and caspase-8.

### Ataxia-Telangiectasia Mutated kinase (ATM) is a pinostrobin-responsive protein

**Figure 2:**
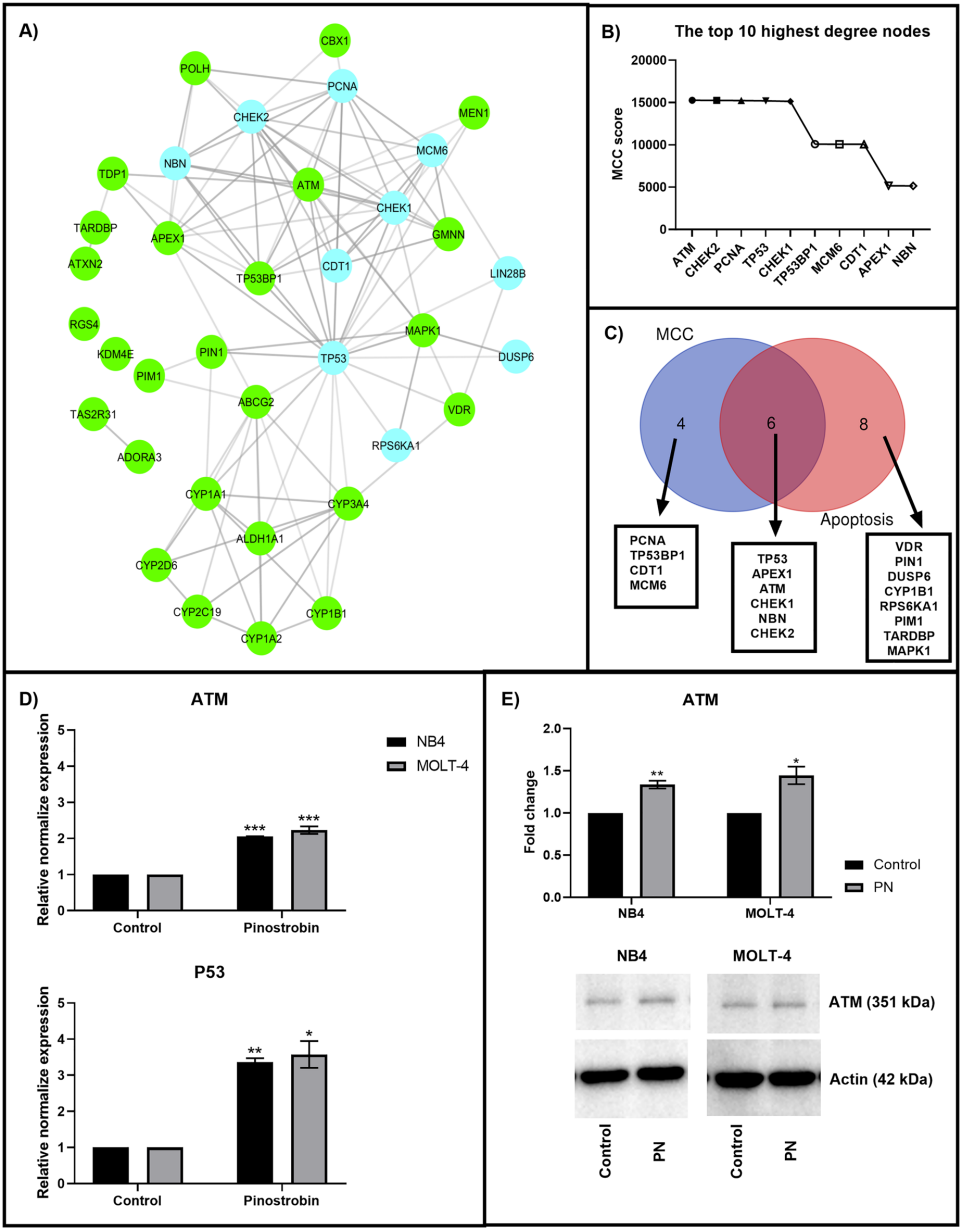
ATM was a PN-responsive protein involved in apoptosis induction in acute leukemia cells. (**A**) The PN-modulated PPIs network was obtained from the STRING database and visualized by Cytoscape software. It must be noted that edges indicate interactions, while green nodes represent potential target proteins predicted from the ChEMBL database and blue nodes represent proteins added from the STRING database. (**B**) The top 10 nodes according to the MCC score were determined by the Cytoscape software. (**C**) The list of top 10 proteins by MCC score, the list of proteins involved in apoptosis, and the list of proteins that were at the intersection of these two lists were all displayed in the Venn diagram. (**D**) The association between the exposure to PN and the mRNA expression levels of ATM and p53 was examined using RT-qPCR. (**E**) The protein expression level of ATM was determined by western blot analysis. Uncropped blots were included in Supplement 3. Data was expressed as the mean ± SEM of three independent experiments. *P < 0.05, **P < 0.01 and ***P < 0.001 were the statistically significant differences from the control group.

Using a molecular similarity approach in the ChEMBL database with a threshold of more than 70%, compounds similar to PN were obtained. Due to the similarity principle, 25 target proteins of PN-homologous compounds with bioactivity potency at a concentration less than 150 μM that are expressed in humans were determined as potential targets of PN. These potential targets of PN are displayed in Table 1. Then, these proteins were used for PPI network construction by the STRING database with an intermediate confidence score > 0.4, and the first and second spheres of interaction were 10 and 0, respectively. From network visualization using Cytoscape, the PPI network comprised a total of 110 interactions involving 35 proteins, as presented in Fig. 2A. The hub protein of this network was then examined utilizing the maximal clique centrality (MCC) methods of the CytoHubba application in Cytoscape. Figure 2B displays the top 10 proteins with the highest MCC score, which included ATM, CHEK2, PCNA, TP53, CHEK1, TP53BP1, MCM6, CDT1, APEX1, and NBN. Based on the gene ontology (GO) analysis via the STRING database, 14 proteins, including DUSP6, RPS6KA1, PIM1, PIN1, MAPK1, APEX1, TP53, NBN, CHEK1, CHEK2, ATM, VDR, CYP1B1, and TARDBP, function in the apoptosis process. As shown in Fig. 2C, the hub proteins of this network that most often interacted with other proteins in the network and were involved in the apoptotic process included ATM, TP53, CHEK2, CHEK1, APEX1, and NBN. Among these 6 proteins, ATM exhibited the highest MCC score. This result indicated that ATM might be an important target protein of PN in inducing apoptosis in acute leukemia cells. The KEGG pathway database was then used to identify the ATM downstream signaling pathway. The findings showed that ATM contributed to the apoptotic process by activating the p53 signaling pathway.

**Table 1:** Potential targets of pinostrobin obtained from ChEMBL that met all inclusion criteria.

Target ChEMBL ID	Target name	Identifiers
CHEMBL256	Adenosine A3 receptor	ADORA3
CHEMBL3577	Aldehyde dehydrogenase 1A1	ALDH1A1
CHEMBL1795085	Ataxin-2	ATXN2
CHEMBL5393	ATP-binding cassette sub-family G member 2	ABCG2
CHEMBL1741193	Chromobox protein homolog 1	CBX1
CHEMBL2231	Cytochrome P450 1A1	CYP1A1
CHEMBL3356	Cytochrome P450 1A2	CYP1A2
CHEMBL4878	Cytochrome P450 1B1	CYP1B1
CHEMBL3622	Cytochrome P450 2C19	CYP2C19
CHEMBL289	Cytochrome P450 2D6	CYP2D6
CHEMBL340	Cytochrome P450 3A4	CYP3A4
CHEMBL5542	DNA polymerase eta	POLH
CHEMBL5619	DNA-(apurinic or apyrimidinic site) lyase	APEX1
CHEMBL1293278	Geminin	GMNN
CHEMBL1293226	Lysine-specific demethylase 4D-like	KDM4E
CHEMBL4040	MAP kinase ERK2	MAPK1
CHEMBL2093861	Menin/Histone-lysine *N*-methyltransferase MLL	MEN1
CHEMBL2288	Peptidyl-prolyl cis–trans isomerase NIMA-interacting 1	PIN1
CHEMBL1795091	Regulator of G-protein signaling 4	RGS4
CHEMBL2147	Serine/threonine-protein kinase PIM1	PIM1
CHEMBL3797	Serine-protein kinase ATM	ATM
CHEMBL2362981	TAR DNA-binding protein 43	TARDBP
CHEMBL2034804	Taste receptor type 2-member 31	TAS2R31
CHEMBL1075138	Tyrosyl-DNA phosphodiesterase 1	TDP1
CHEMBL1977	Vitamin D receptor	VDR

### ATM and p53 are involved in apoptosis induction by pinostrobin in acute leukemia cells

The effect of PN on ATM and p53 expression was investigated to verify the network analysis findings. Leukemia cells were treated with PN at the IC50 for 48 h, followed by detection of ATM and p53 expression via RT‒qPCR and western blot analysis. The results shown in Fig. 2D revealed that PN increased the mRNA expression levels of ATM and p53 in both leukemia cell lines. These results were consistent with the western blot results shown in Fig. 2E, which similarly demonstrated the elevation of ATM after 48 h of PN treatment. These findings supported the hypothesis that PN can promote apoptosis by upregulating ATM, which further activates the p53 signaling pathway.

### miR-181b-5p is predicted to be an ATM-specific miRNA

**Figure 3:**
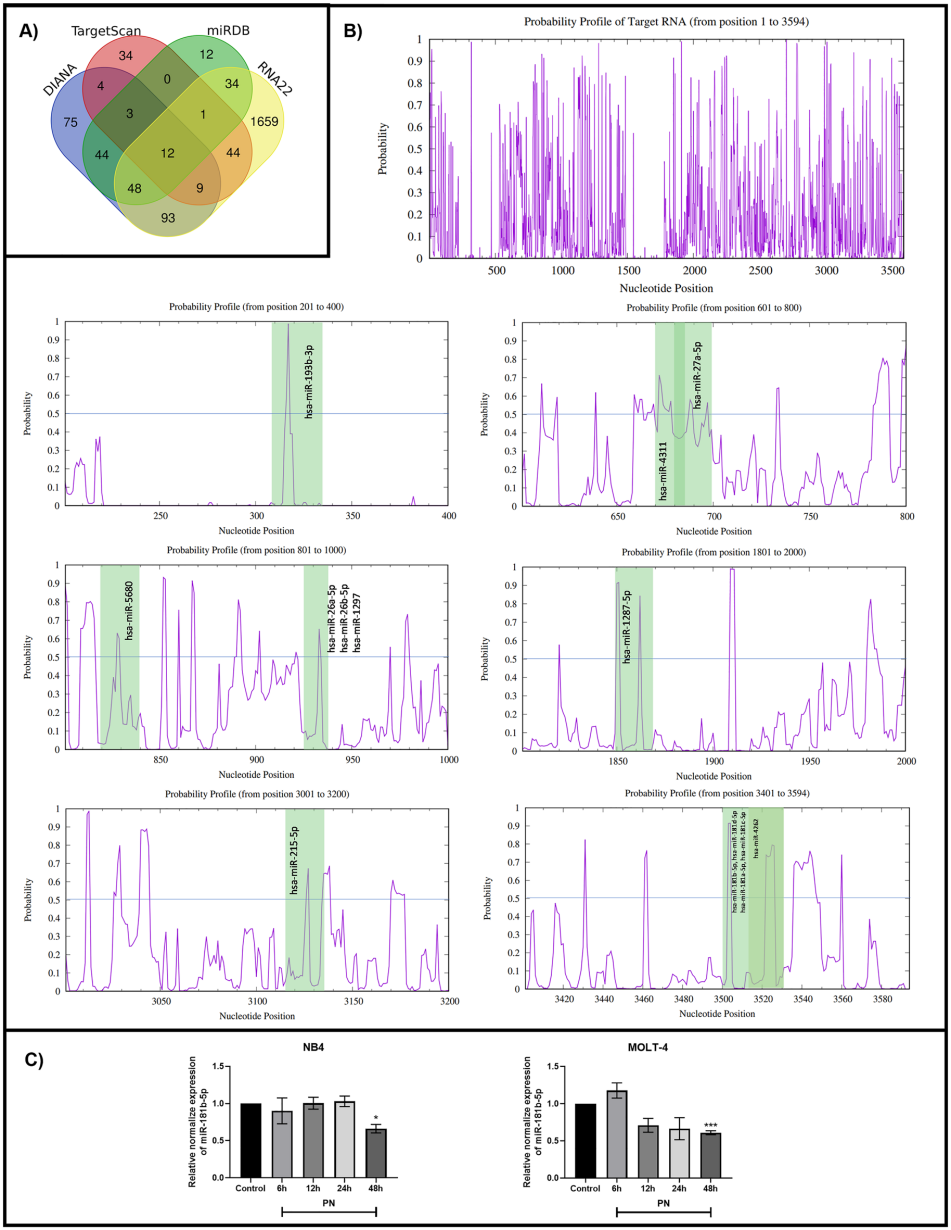
miR-181b-5p was an ATM-regulated miRNA that was predicted via a bioinformatics approach. (**A**) The Venn diagram demonstrated the respective numbers of miRNAs that were obtained from 4 prediction tools, including DIANA, miRDB, TargetScanHuman and RNA22. (**B**) The purple histogram was the probability profile of ATM mRNA. The green band represented the binding site of each miRNA. (**C**) The expression level of miR-181b-5p after treatment with the IC50 concentration of PN was examined using RT-qPCR. Data was expressed as the mean ± SEM of three independent experiments. *P < 0.05, **P < 0.01 and ***P < 0.001 were the statistically significant difference from the control group.

The miRNAs that regulate ATM were predicted by four different prediction tools. There were 288, 154, 107, and 1900 miRNAs obtained from DIANA, miRDB, TargetScanHuman, and RNA22, respectively. In accordance with Fig. 3A, 73 miRNAs, consisting of 12 miRNAs retrieved from all prediction tools and 51 miRNAs predicted from three prediction tools, were obtained. Next, the miRNAs were manually selected based on the selection criteria described in the materials and methods. Overall, 22 possible ATM-regulated miRNAs met all the criteria. The target accessibility of each possible ATM-regulated miRNA was evaluated using Sfold. From a probability histogram in Fig. 3B, positions of nucleotides with a probability greater than 0.5 were defined as accessible regions for miRNA binding. Fourteen candidate miRNAs for ATM suppression, listed in Table 2, could hybridize to the accessible regions of ATM mRNA. These miRNAs were then reviewed for their supportive evidence in inhibiting ATM. Strong supporting evidence provided by RT‒qPCR, western blot, or luciferase test results confirmed the regulation of ATM by miR-26a-5p, miR-26b-5p, miR-27a-5p, miR-181a-5p, and miR-181b-5p^23–28^. Among the 5 miRNAs, miR-181b-5p was chosen because it had the lowest ∆G hybrid value and the highest LogitProb value.

**Table 2:** The list of potential ATM-specific miRNA candidates.

miRNA	Site position	LogitProb*	∆G hybrid**
hsa-miR-193b-3p	308–336	0.773477	− 23.6
hsa-miR-4311	671–685	0.891580	− 17.7
hsa-miR-27a-5p	680–699	0.781724	− 19.1
hsa-miR-5680	819–839	0.858980	− 18
hsa-miR-26a-5p	926–938	0.738008	− 17.4
hsa-miR-26b-5p	926–938	0.738008	− 17.4
hsa-miR-1297	928–938	0.695976	− 15.1
hsa-miR-1287-5p	1847–1869	0.701477	− 19.9
hsa-miR-215-5p	3116–3135	0.710925	− 21.2
hsa-miR-181a-5p	3504–3531	0.849549	− 16.9
hsa-miR-181c-5p	3504–3531	0.834222	− 14.6
hsa-miR-181b-5p	3505–3531	0.863551	− 20.5
hsa-miR-181d-5p	3505–3531	0.853119	− 18.6
hsa-miR-4262	3516–3531	0.851064	− 15.2

*The LogitProb value represents the likelihood of binding to the target site of mRNA.

**The ∆G hybrid value reflects the stability of the miRNA-target hybrid.

### miR-181b-5p is downregulated in pinostrobin-treated leukemia cells

To verify the result from the prediction of ATM-regulated miRNAs, the expression level of miR-181b-5p was investigated via RT‒qPCR. NB4 and MOLT-4 cells were treated with the IC50 of PN for 6, 12, 24, and 48 h. The results presented in Fig. 3C demonstrate that PN significantly suppressed the expression level of miR-181b-5p at 48 h in NB4 and MOLT-4 cells. This result indicated that miR-181b-5p is involved in the mechanism of PN-induced apoptosis in acute leukemia cells.

### miR-181b-5p is involved in PN-induced apoptosis in acute leukemia cells

**Figure 4:**
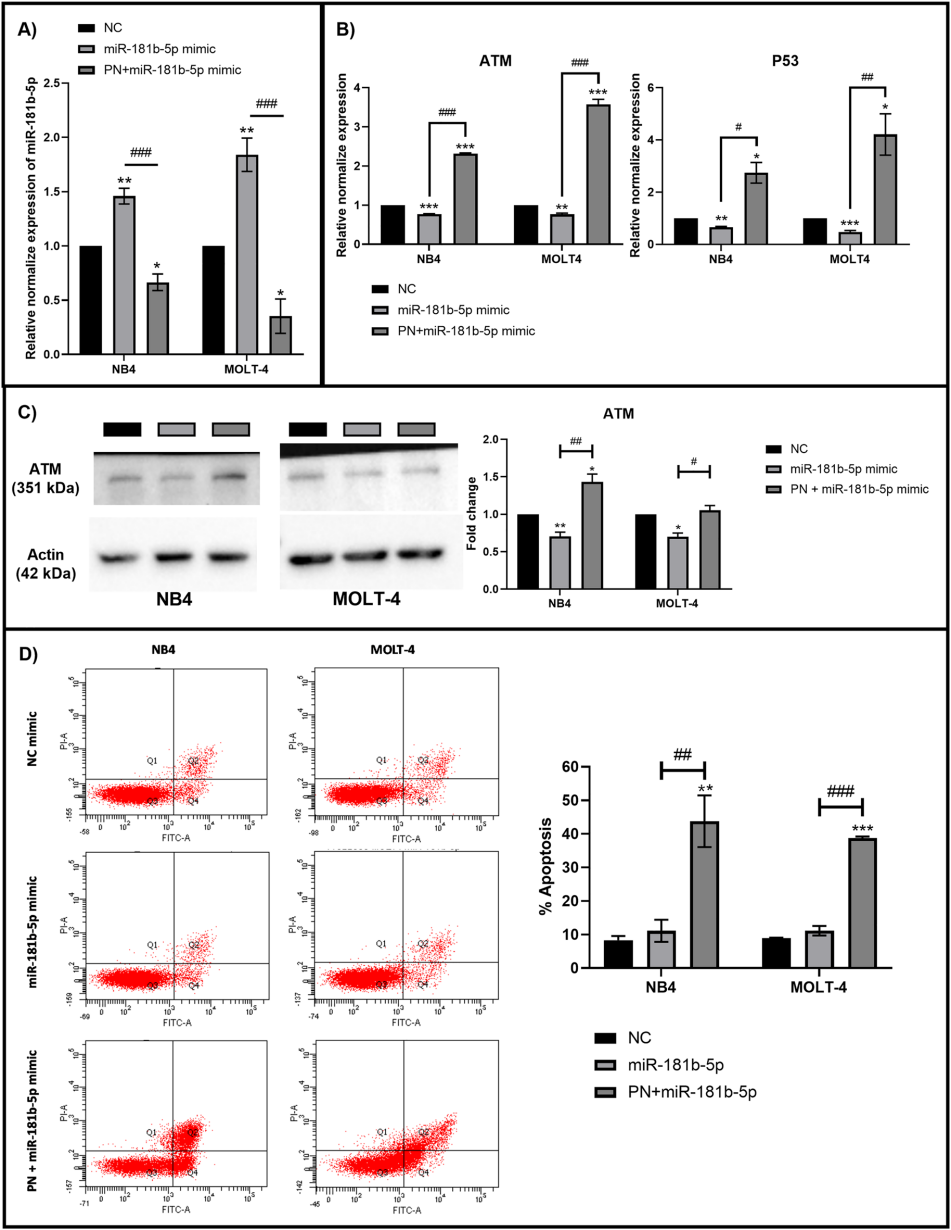
miR-181b-5p played an important role in the activation of apoptosis in acute leukemia cells by pinostrobin. (**A**) The expression of miR-181b-5p following miR-181b-5p mimic transfection alone and in combination with PN was determined by RT-qPCR. (**B**) RT-qPCR was used to assess the expression of ATM and p53 after miR-181b-5p mimic transfection alone and in combination with PN. (**C**) After miR-181b-5p mimic transfection, the expression of ATM was examined using western blot analysis. Uncropped blots were included in a Supplement 4. (**D**) Using Annexin V-FITC/PI labeling and cytometric analysis, the percentage of apoptosis was measured. Data was expressed as the mean ± SEM of three independent experiments. *P < 0.05, **P < 0.01 and ***P < 0.001 were the statistically significant difference from the control group. ^#^P < 0.05, ^##^P < 0.01 and ^###^P < 0.001 were the statistically significant difference from the miR-181b-5p mimic transfected group.

The miR-181b-5p mimic was transiently transfected into leukemia cells alone and in combination with PN at the IC50 to determine whether miR-181b-5p expression levels have any influence on the expression of ATM and p53 and the activation of apoptosis. Leukemia cells transfected with the microRNA mimic DS control served as the control group. To determine the efficiency of the transfection processes, the expression of miR-181b-5p was investigated using RT‒qPCR. The results in Fig. 4A demonstrate that the expression of miR-181b-5p was increased in cells transfected with miR-181b-5p mimics alone compared to the control group. However, the expression of miR-181b-5p in cells that were transfected with miR-181b-5p mimic followed by PN treatment was decreased. The results showed that in miR-181b-5p-overexpressing cells, PN was still effective in inhibiting miR-181b-5p expression. Then, to examine the regulatory effect of miR-181b-5p on ATM and p53, transfected cells under different conditions were harvested to measure ATM and p53 expression levels using RT‒qPCR. Figure 4B shows that ATM and p53 were repressed in miR-181b-5p-mimic-transfected cells. This outcome supported the role of miR-181b-5p in ATM inhibition. The expression of ATM and p53 increased after miR-181b-5p mimic transfection with PN therapy. These results implied that PN might silence miR-181b-5p to activate the ATM and p53 signaling pathways. The expression of ATM at the mRNA level also correlated with its expression at the protein level, which was investigated by western blot analysis (Fig. 4C). The percentage of apoptosis was determined using Annexin V-FITC and PI staining to study the impact of miR-181b-5p on apoptosis induction. The percentage of apoptosis in miR-181b-5p-transfected cells was not different from that in the control group, as shown in Fig. 4D. However, the percentage of apoptosis increased in cells that were transfected with miR-181b-5p mimic followed by PN treatment. These results suggested that PN induces apoptosis in leukemia cells by regulating miR-181b-5p expression.

## Discussion

Pinostrobin is a major phytochemical in the group of flavonoids found in fingerroot (*Boesenbergia rotunda*)^8^. In this study, we explored the anticancer properties of PN in NB4 and MOLT-4 acute leukemia cells. PN reduced the viability of both leukemia cell lines via apoptosis induction, resulting in an increase in the proportion of cells in the sub-G1 phase of the cell cycle and the percentage of apoptotic cells. These results correlated with the outcomes of previous studies that investigated the anticancer activities of PN in acute leukemia (Jurkat and HL60)^14^, chronic leukemia (K562) cells^12^, and other types of cancer, including cervical cancer^13^, breast cancer and glioblastoma^12^. Although there is evidence of the anti-leukemic activity of PN, the underlying mechanism of its role in apoptosis induction is still unclear. Therefore, the effect of PN on apoptotic gene expression was investigated. Caspase-8 and caspase-10 are engaged in the extrinsic apoptotic pathway, while caspase-9 triggers the intrinsic apoptotic pathway. The activation of these initiator caspases further activates caspase-3, leading to apoptosis^29^. Our results revealed that caspase-3, caspase-8, and caspase-9 were upregulated in PN-treated cells compared to control cells. Moreover, PN was shown to increase the expression of BAX and Fas, a proapoptotic protein that plays a role in the intrinsic pathway of apoptosis and a death receptor that is important for the extrinsic apoptotic pathway, respectively. These findings indicated that PN induces apoptosis in acute leukemia cells via the intrinsic and extrinsic apoptotic pathways by activating the caspase family (caspase-3, 8, and 9), pro-apoptotic protein (BAX), and death receptor (Fas). A toxicity study of PN was performed in Wistar rats, demonstrating that PN is nontoxic at doses between 1 and 100 mg/kg^30^. According to previous studies, our research also revealed that PN had a lesser effect on PBMCs from healthy subjects.

To further understand the underlying mechanism of PN-mediated apoptosis, the target protein of PN was identified using the ligand-based approach and the PPI network. We obtained 25 potential target proteins of PN from the ChEMBL database via the similarity principle. These proteins, together with 10 others from the STRING database, were used to construct the PPI network. Among these 35 proteins, 14 function in the apoptosis process. The MCC method can be used to identify essential proteins from complex interactomes^31^. Because ATM had the highest MCC score, it might be the key target protein of PN in apoptosis induction. According to the findings of the KEGG pathway study as well as other research^32^, ATM has been demonstrated to mediate the activation of the apoptotic pathway in response to DNA damage. Many previous studies reported that ATM phosphorylates p53 to stabilize p53, and then the phosphorylated p53 will further activate the expression of a panel of apoptotic genes and facilitate both intrinsic and extrinsic apoptotic pathways. Recently, in response to DNA damage, ATM can promote the expression of p53 mRNA and the synthesis of p53 protein by phosphorylating MDM2 at Ser395. This phosphorylation brings MDM2 to stabilize the p53 mRNA and recruits ATM to the p53 polysome to phosphorylate the nascent p53 protein and prevents newly synthesized p53 from MDM2-mediated degradation^33,34^. To verify the prediction from the PPI network, the expression of ATM and p53 was determined using RT‒qPCR and western blot analysis. NB4 and MOLT-4 displayed overexpression of ATM and p53 after PN treatment. These results suggest that PN can promote intrinsic and extrinsic apoptotic pathways by upregulating ATM, which further activates the p53 signaling pathway. Several studies have reported that the p53 signaling pathway and ATM involved in apoptosis can be induced by natural compounds. For example, the fungal metabolite galiellalactone induces cell cycle arrest and apoptosis in prostate cancer cells via ATM phosphorylation^35^. Bavachinin activates ATM to induce apoptosis in small cell lung cancer cells^36^. In addition, curcumin, an active ingredient of turmeric, shows antitumor activity by targeting the ATM/Chk2/p53 signaling pathway^37^.

MicroRNAs have been mentioned as epigenetic factors controlling the posttranscriptional regulation of gene expression. They control a wide range of cellular functions, including growth, proliferation, and degeneration. Recently, the involvement of miRNAs in phytochemical-mediated apoptosis has received much attention^16^. We applied bioinformatics methods from Seenprachawong et al.^38^ to predict miRNAs related to PN-induced apoptosis in acute leukemia. One miRNA discovered using bioinformatics was miR-181b-5p, a member of the miR-181 family, which is highly evolutionarily conserved in almost all vertebrates. This miRNA family consists of four members, including miR-181a, miR-181b, miR-181c, and miR-181d^39^. Many studies have demonstrated that the miR-181 family regulates the differentiation of granulocytic and macrophage-like cells by targeting PRKCD, CTDSPL, and CAMKK. The miR-181 family was also found to be a marker of common myeloid and erythroid progenitor commitment^40^. There is evidence that miR-181 is overexpressed in several malignancies, including acute myeloid leukemia, colorectal cancer, and breast cancer^41–43^. Our study showed that miR-181b-5p acts as an oncogene by regulating ATM and p53. By transiently transfecting the miR-181b-5p mimic into acute leukemia cells, the expression of ATM and p53 was repressed. To the contrary, the reduction of miR-181b-5p by PN increased the expression of ATM. These results were consistent with previous studies from Yujun Wang and Andrea BISSO^23,44^. They showed that the ectopic overexpression and inhibition of miR-181a/b, respectively, were related to the decreased and enhanced expression of ATM at both the mRNA and protein levels. Also, they used a luciferase reporter assay to confirm that the 3′ UTR of ATM was a direct target of miR-181a/b. Moreover, previous studies revealed that overexpression of the miR-181 family showed a strong correlation with overall survival and aggressiveness of cancers, including leukemia^23,41^.

The present study demonstrated the anti-leukemic effects of PN on NB4 and MOLT-4 cell lines since PN can suppress miR-181b-5p, increase the expression of ATM and p53, and trigger apoptosis in acute leukemia cells, including those with ectopically expressed miR-181b-5p. These results highlighted the ability of PN to be used therapeutically for leukemia in the future. Additionally, these findings may be beneficial for improving our understanding of the role of miR-181b-5p in human leukemia.

## Methods

### Leukemia cell culture

The human acute promyelocytic leukemia cell line (NB4) and the human acute lymphocytic leukemia cell line (MOLT-4) were purchased from Cell Lines Service (Eppelheim, Germany). Leukemia cells were cultured in RPMI-1640 medium supplemented with 10% (v/v) FBS and 1% (v/v) penicillin–streptomycin (Gibco Life Technologies, Waltham, MA, USA). Cells were maintained in a humidified incubator at 37 °C with 5% CO_2_. The medium was changed every 2–3 days.

### Isolation of peripheral blood mononuclear cells (PBMCs)

With the ethical approval of the Mahidol University Central Institution Review Board (MU-CIRB) (No. MU-CIRB 2021/422.0110), all experiments were performed in accordance with relevant guidelines and regulations. Prior to blood collection, healthy donors provided written informed consent. Then, 10 ml of heparinized blood samples from healthy donors were diluted with PBS at a 1:1 ratio and carefully overlayered over the LymphoprepTM solution (Alere Technology AS, Oslo, Norway). After centrifugation at 800×*g* for 20 min, PBMCs were collected. Before being applied in the cell viability assay, PBMCs were washed twice with RPMI-1640 media.

### Determination of cell viability by MTT assay

Leukemia cells (1.5 × 10^4^ cells) and isolated PBMCs (1 × 10^5^ cells) were treated with 100, 200, and 300 µM of PN (Sigma-Aldrich, Schnell Dorf, Germany), which was dissolved in 1.5% DMSO for 24 and 48 h. For the control group, cells were treated with the 1.5% DMSO medium without PN. Then, 10 µl of 5 mg/ml MTT solution (Thermo Fisher Scientific, Inc., Waltham, MA, USA) was added to the cells. After incubation for 4 h, the formazan crystal was dissolved by overnight incubation with 100 µl of 10% SDS in 0.01 M HCl. The absorbance of each well was measured by a microplate reader at a single wavelength of 570 nm. The half-maximal inhibitory concentration (IC50) was calculated by GraphPad Prism version 8.0 with nonlinear regression analysis (GraphPad Inc., San Diego, CA, USA).

### Investigation of the cell cycle by flow cytometry

Cells (1 × 10^5^ cells) cultured in the medium with or without PN were harvested and washed twice with PBS. The fixation was performed by incubating the cells with 70% ethanol for 30 min at 4 °C. After washing the fixed cells with PBS, the cells were incubated with 100 µg/ml RNase A (Merck Millipore, Burlington, MA, USA) for 30 min at 37 °C. Following that, 50 g/ml PI (BD Biosciences, Palo Alto, CA, USA) was added and incubated for 10 min in the dark at 4 °C. The cells were examined by a FACSCantoII flow cytometer and analyzed using the FACSDiva software (BD Biosciences, Palo Alto, CA, USA).

### Analysis of cell apoptosis by Annexin V-FITC and PI staining

Apoptosis-mediated cell death was examined by the FITC Annexin V apoptosis detection kit (BD Biosciences, Palo Alto, CA, USA) according to the manufacturer’s instructions. In brief, cells were harvested and washed twice with 1X PBS, then resuspended in 100 µl of Annexin V binding buffer. After that, cells were stained with 5 µl of Annexin V-FITC and 5 µl of PI at room temperature in a darkened container for 15 min. After incubation, 400 µl of Annexin V binding buffer was added. Using a FACSCantoII flow cytometer and the FACSDiva software, apoptotic cells were discovered.

### PPI network construction and Identification of PN-responsive proteins

Compounds sharing more than 70% of a chemical structure similar to PN were obtained from the ChEMBL database (https://www.ebi.ac.uk/chembl/)^45^. Based on the similarity principle, structurally similar compounds may share common targets. Therefore, targets of PN-homologous compounds may likewise be potential targets of PN. Then, these discovered target proteins were chosen for PPI network construction to observe how these proteins interact and identify the most probable target of PN in the induction of apoptosis. The chosen proteins must be expressed in *Homo sapiens* and are target proteins for substances with bioactivity potency at concentrations less than 150 μM. The PPI network was constructed by the STRING database (http://string-db.org)^46^. Protein and gene interaction networks in biological organisms are frequently scale-free networks that obey the power law of P(k) ~ k^−γ^^47^. In order to obtain a scale-free network which is an optimal network, the network was set up with an intermediate confidence score of more than 0.4 and the first and second spheres of interaction were varied from 10 to 100 nodes. All networks were exported to Cytoscape software to analyze network topology^48,49^. The γ and R-square in correlation with the power law of all networks were listed as Supplement 1. The highest R-squared was found in the network with 10 first sphere nodes and no second sphere nodes. As a result, this network was chosen for further investigation. A gene ontology (GO) analysis of this network was performed by the STRING database to study the underlying mechanism of PN in the induction of apoptosis. The PN-responsive protein was identified using the Maximal Clique Centrality (MCC) calculation method of the CytoHubba application in Cytoscape^31^. After that, the downstream signaling pathway of PN-responsive protein was obtained from the KEGG pathway database (https://www.genome.jp/kegg/pathway.html)^50^.

### MicroRNA prediction

A 3′UTR sequence of Ataxia-telangiectasia mutated kinase (ATM) mRNA, a PN-responsive protein, was obtained from GenBank (https://www.ncbi.nlm.nih.gov/nuccore/NM_000051.4?report=genbank)^51^ by searching accession number as NM_000051. Four widely used web-based prediction tools, including RNA22 (https://cm.jefferson.edu/rna22/)^52^, TargetScanHuman (http://www.targetscan.org/vert_72/)^53^, DIANA-microT-CDS (www.microrna.gr)^54^, and miRDB (http://mirdb.org)^55^, in an updated version, were used for predicting miRNAs that target ATM. Only miRNAs that (1) were predicted by more than or equal to three prediction tools; (2) had a p-value less than 0.05 if obtained from RNA22; (3) had a prediction score greater than 80 if obtained from miRDB; (4) had a miTG score greater than or equal to 0.7 if obtained from DIANA-tools; and (5) had a context + score less than or equal to-0.1 or P_CT_ greater than or equal to 0.5 if obtained from TargetScanHuman were selected. The miRNAs were then examined using Sfold (http://sfold.wadsworth.org/starmir.html) to determine their target accessibility^56^. A sequence of the 3′UTR of the ATM mRNA was used as an input to obtain a probability histogram. Since a high probability will increase the chance of successful miRNA binding, the particular positions of nucleotides with a probability greater than 0.5 were defined as an accessible region for miRNA^38^. miRNAs that could hybridize with the accessible region were categorized as candidate miRNAs for inhibition of the ATM gene. The candidate miRNAs were reviewed for their supportive evidence in targeting ATM before being selected for further experiments.

### miR-181b-5p mimics transfection

1 h before transfection, 2 × 10^5^ leukemia cells in 450 μl of RPMI-1640 were seeded into a 24-well plate. A commercial miR-181b-5p mimic (GenePharma, Shanghai, China) with the following sequence: 5′-AACAUUCAUUGCUGUCGGUGGGU-3′ was used for transfection. The transfection was achieved using the lipofectamine 3000™ (Invitrogen, Carlsbad, CA, USA) according to the manufacturer’s instructions. Briefly, 1.5 μl of lipofectamine 3000™ was diluted in 25 μl of serum-free Opti-MEM™ medium (Gibco Life Technologies, Waltham, MA, USA). The miR-181b-5p mimic was diluted to a concentration of 1 μM using serum-free Opti-MEM™ medium as a diluent. Then, the diluted miR-181b-5p mimic was mixed with the diluted lipofectamine 3000™ at a 1:1 ratio (v/v). After incubation for 15 min at room temperature, 50 μl of the mimic-lipofectamine mixture was added to leukemia cells to a final concentration of 50 nM miR-181b-5p mimic. Cells were further incubated for 48 h prior to harvesting for PN treatment. The miR-181 transfected cells were treated with the IC50 of PN and 1.5% DMSO for 48 h. Then, cells were harvested to measure the miR-181b-5p, ATM and p53 expression levels using RT-qPCR, investigate the expression of ATM by western blot analysis and assess apoptosis mediated cell death using Annexin V-FITC and PI staining. Leukemia cells transfected with the microRNA mimic DS control (GenePharma, Shanghai, China) for 48 h followed by incubation with 1.5% DMSO for 48 h were served as a negative control.

### Determination of gene expression and miR-181b-5p expression by reverse transcription-quantitative PCR (RT-qPCR)

Cells were harvested by centrifugation at 3500 rpm for 5 min. Total RNA was extracted using the GENEzol™ reagent (New England Biolab, Inc., Ipswich, MA, USA). For miRNA, RNA was extracted using Direct-zol RNA Miniprep Kits (Zymo Research, Irvine, CA, USA) according to the manufacturer’s instructions. RNA concentration was measured by the Nanodrop spectrophotometer (Thermo Scientific, Waltham, MA, USA). The Thermo Scientific RevertAid first-strand cDNA synthesis kit (Thermo Scientific, Waltham, MA, USA) was used to synthesize cDNA for investigating mRNA expression while the miRNA 1st-strand cDNA Synthesis Kit (Agilent Technologies, Santa Clara, CA, USA) was used to synthesize cDNA for miRNA expression. The primers were designed from literature reviews and their quality was determined by the Primer-BLAST^57^ and the BLASTN database^58^. The primer sequences used in this study are showed in Supplement 2. Then, qPCR was performed using the designed primers, Luna^®^ Universal qPCR Master Mix (New England Biolab, Inc., Ipswich, MA, USA) and CFX Connect Real-Time PCR Detection System (Bio-Rad, Inc., Hercules, CA, USA). The relative mRNA and miRNA expression of target genes were analyzed by Bio-ad CFX Manager software (Bio-Rad, Inc., Hercules, CA, USA) using the 2^−∆∆CT^ method^59^. GAPDH was used to normalize mRNA expression and U6 was used to normalize miRNA expression.

### Western blot analysis

After being harvested, the cells underwent two 1X PBS washes. The whole cell lysate was prepared by incubating the cells for 15 min in 0.1 ml of cold RIPA lysis buffer containing 1% protease inhibitor, followed by centrifugation at 14,000×*g* for 10 min at 4 °C. The Bio-Rad Protein Assay Dye Reagent Concentrate (Bio-Rad, Inc., Hercules, CA, USA) was used to measure the protein concentration in accordance with the manufacturer's instructions. Then, equal amounts of proteins (10 μg) were separated by sodium dodecyl sulfate–polyacrylamide gel electrophoresis (SDS–PAGE) and transferred to nitro cellulose membranes. Following that, membranes were incubated at 4 °C for an overnight period with monoclonal mouse anti-ATM and anti-β-actin. The membranes were then immunoblotted with HRP-conjugated horse anti-mouse IgG antibody at 37 °C for 90 min. The signal was developed with an enhanced chemiluminescence (ECL) substrate and detected chemiluminescence by the ChemiDoc™ MP Imaging System. Band density was quantitated using the Image Lab™ software.

### Statistical analysis

Three duplicates of each experiment were carried out. The findings were displayed as mean ± standard error of mean (SEM). Every statistical analysis was done using SPSS (SPSS Inc., Chicago, IL, USA). The student t-test was used to compare the results between the two groups, and one-way ANOVA was used to compare the results between more than two groups. Statistical significance was defined as a p-value of less than 0.05.

## Supplementary Information

Supplementary Information.

## Data Availability

The datasets generated and/or analyzed during the current study are available in the GenBank repository, [Accession number: NM_000051, https://www.ncbi.nlm.nih.gov/nuccore/NM_000051.4?report=genbank]. Further information and requests for resources and reagents should be directed to and will be fulfilled by the Lead Contact Dalina Tanyong (dalina.itc@mahidol.ac.th).
