# Supplementary material for: Pinostrobin, a fingerroot compound, regulates miR-181b-5p and induces acute leukemic cell apoptosis
Source: Sci Rep. 2023 May 19;13:8084. doi: 10.1038/s41598-023-35193-6 (PMC10198993; doi:10.1038/s41598-023-35193-6)

**Supplementary table 1:** Topology parameter calculated by Cytoscape in each network. The yellow highlight was selected as the optimal parameters.

|  |  |  |  |  | Indirected network analysis | | | |
| --- | --- | --- | --- | --- | --- | --- | --- | --- |
| Network No. | 1st shell | 2nd shell | nodes | edges | Clustering coefficient | b (-Ƴ) | Correlation | R-squared |
| 1 | 0 | 0 | 25 | 40 | 0.293 | 0.538 | 0.274 | 0.176 |
| 2 | 10 | 0 | 35 | 110 | 0.577 | -0.267 | 0.437 | 0.302 |
| 3 | 10 | 10 | 45 | 228 | 0.626 | -0.027 | 0.034 | 0.006 |
| 4 | 10 | 20 | 55 | 421 | 0.664 | -0.049 | 0.152 | 0.020 |
| 5 | 10 | 30 | 65 | 664 | 0.691 | -0.028 | 0.115 | 0.013 |
| 6 | 10 | 40 | 75 | 917 | 0.698 | 0.041 | 0.181 | 0.038 |
| 7 | 10 | 50 | 85 | 1228 | 0.701 | 0.033 | 0.143 | 0.026 |
| 8 | 10 | 60 | 95 | 1673 | 0.718 | 0.008 | 0.004 | 0.002 |
| 9 | 10 | 70 | 105 | 2146 | 0.714 | 0.001 | -0.054 | 0.000 |
| 10 | 10 | 80 | 115 | 2496 | 0.708 | 0.009 | 0.043 | 0.004 |
| 11 | 10 | 90 | 125 | 2918 | 0.706 | 0.021 | 0.025 | 0.010 |
| 12 | 10 | 100 | 135 | 3302 | 0.711 | -0.027 | 0.204 | 0.026 |
| 13 | 20 | 0 | 45 | 225 | 0.635 | -0.028 | 0.034 | 0.007 |
| 14 | 20 | 10 | 55 | 398 | 0.662 | 0.021 | -0.032 | 0.003 |
| 15 | 20 | 20 | 65 | 623 | 0.677 | -0.009 | 0.079 | 0.001 |
| 16 | 20 | 30 | 75 | 905 | 0.700 | -0.012 | 0.098 | 0.003 |
| 17 | 20 | 40 | 85 | 1260 | 0.713 | -0.003 | 0.060 | 0.000 |
| 18 | 20 | 50 | 95 | 1630 | 0.714 | 0.006 | -0.015 | 0.001 |
| 19 | 20 | 60 | 105 | 2088 | 0.719 | -0.014 | 0.125 | 0.008 |
| 20 | 20 | 70 | 115 | 2621 | 0.714 | 0.003 | -0.028 | 0.000 |
| 21 | 20 | 80 | 125 | 3060 | 0.717 | 0.013 | 0.030 | 0.007 |
| 22 | 20 | 90 | 135 | 3580 | 0.714 | 0.014 | 0.031 | 0.007 |
| 23 | 20 | 100 | 145 | 3852 | 0.709 | -0.011 | 0.146 | 0.002 |
| 24 | 30 | 0 | 55 | 417 | 0.682 | 0.002 | 0.017 | 0.000 |
| 25 | 30 | 10 | 65 | 643 | 0.689 | 0.004 | 0.004 | 0.000 |
| 26 | 30 | 20 | 75 | 913 | 0.696 | 0.013 | -0.021 | 0.002 |
| 27 | 30 | 30 | 85 | 1219 | 0.702 | 0.051 | 0.151 | 0.039 |
| 28 | 30 | 40 | 95 | 1556 | 0.715 | 0.016 | 0.007 | 0.006 |
| 29 | 30 | 50 | 105 | 1820 | 0.709 | -0.031 | 0.178 | 0.022 |
| 30 | 30 | 60 | 115 | 2187 | 0.711 | -0.015 | 0.104 | 0.006 |
| 31 | 30 | 70 | 125 | 2595 | 0.703 | -0.014 | 0.105 | 0.005 |
| 32 | 30 | 80 | 135 | 3104 | 0.702 | -0.015 | 0.111 | 0.005 |
| 33 | 30 | 90 | 145 | 3567 | 0.692 | -0.011 | 0.099 | 0.003 |
| 34 | 30 | 100 | 155 | 3968 | 0.691 | -0.021 | 0.140 | 0.011 |
| 35 | 40 | 0 | 65 | 577 | 0.699 | 0.044 | 0.171 | 0.023 |
| 36 | 40 | 10 | 75 | 820 | 0.706 | 0.060 | 0.224 | 0.045 |
| 37 | 40 | 20 | 85 | 1090 | 0.701 | 0.020 | 0.020 | 0.006 |
| 38 | 40 | 30 | 95 | 1420 | 0.702 | 0.000 | 0.072 | 0.000 |
| 39 | 40 | 40 | 105 | 1754 | 0.704 | 0.001 | -0.054 | 0.000 |
| 40 | 40 | 50 | 115 | 2151 | 0.713 | 0.002 | -0.063 | 0.000 |
| 41 | 40 | 60 | 125 | 2505 | 0.719 | -0.025 | 0.158 | 0.016 |
| 42 | 40 | 70 | 135 | 2926 | 0.714 | 0.002 | 0.008 | 0.000 |
| 43 | 40 | 80 | 145 | 3341 | 0.707 | -0.007 | 0.050 | 0.001 |
| 44 | 40 | 90 | 155 | 3815 | 0.700 | -0.003 | 0.052 | 0.000 |
| 45 | 40 | 100 | 165 | 4329 | 0.689 | -0.025 | 0.151 | 0.014 |
| 46 | 50 | 0 | 75 | 813 | 0.711 | 0.028 | 0.044 | 0.008 |
| 47 | 50 | 10 | 85 | 1061 | 0.707 | 0.030 | 0.046 | 0.010 |
| 48 | 50 | 20 | 95 | 1377 | 0.708 | 0.015 | -0.008 | 0.003 |
| 49 | 50 | 30 | 105 | 1726 | 0.705 | 0.025 | 0.051 | 0.011 |
| 50 | 50 | 40 | 115 | 2091 | 0.703 | -0.001 | 0.029 | 0.000 |
| 51 | 50 | 50 | 125 | 2478 | 0.709 | -0.007 | 0.054 | 0.001 |
| 52 | 50 | 60 | 135 | 2812 | 0.712 | -0.041 | 0.227 | 0.045 |
| 53 | 50 | 70 | 145 | 3231 | 0.706 | -0.016 | 0.092 | 0.007 |
| 54 | 50 | 80 | 155 | 3583 | 0.699 | -0.002 | 0.026 | 0.000 |
| 55 | 50 | 90 | 165 | 4057 | 0.693 | -0.010 | 0.074 | 0.002 |
| 56 | 50 | 100 | 175 | 4621 | 0.687 | -0.005 | 0.051 | 0.001 |
| 57 | 60 | 0 | 85 | 1081 | 0.717 | 0.030 | 0.125 | 0.016 |
| 58 | 60 | 10 | 95 | 1345 | 0.710 | 0.017 | 0.055 | 0.004 |
| 59 | 60 | 20 | 105 | 1691 | 0.707 | 0.000 | 0.310 | 0.000 |
| 60 | 60 | 30 | 115 | 2047 | 0.703 | 0.008 | 0.014 | 0.001 |
| 61 | 60 | 40 | 125 | 2406 | 0.702 | -0.009 | 0.060 | 0.002 |
| 62 | 60 | 50 | 135 | 2696 | 0.693 | -0.034 | 0.189 | 0.030 |
| 63 | 60 | 60 | 145 | 3138 | 0.695 | -0.033 | 0.203 | 0.033 |
| 64 | 60 | 70 | 155 | 3539 | 0.696 | -0.026 | 0.136 | 0.016 |
| 65 | 60 | 80 | 165 | 3954 | 0.694 | -0.023 | 0.146 | 0.013 |
| 66 | 60 | 90 | 175 | 4246 | 0.686 | -0.026 | 0.155 | 0.017 |
| 67 | 60 | 100 | 185 | 4726 | 0.680 | -0.027 | 0.167 | 0.020 |
| 68 | 70 | 0 | 95 | 1281 | 0.701 | 0.070 | 0.218 | 0.055 |
| 69 | 70 | 10 | 105 | 1524 | 0.701 | 0.045 | 0.138 | 0.018 |
| 70 | 70 | 20 | 115 | 1731 | 0.690 | 0.045 | 0.165 | 0.020 |
| 71 | 70 | 30 | 125 | 2028 | 0.690 | 0.033 | 0.092 | 0.013 |
| 72 | 70 | 40 | 135 | 2388 | 0.691 | 0.007 | -0.005 | 0.001 |
| 73 | 70 | 50 | 145 | 2524 | 0.688 | -0.024 | 0.126 | 0.010 |
| 74 | 70 | 60 | 155 | 2827 | 0.679 | -0.020 | 0.100 | 0.006 |
| 75 | 70 | 70 | 165 | 3127 | 0.684 | -0.036 | 0.175 | 0.023 |
| 76 | 70 | 80 | 175 | 3278 | 0.673 | -0.045 | 0.219 | 0.033 |
| 77 | 70 | 90 | 185 | 3530 | 0.672 | -0.055 | 0.226 | 0.041 |
| 78 | 70 | 100 | 195 | 3937 | 0.672 | -0.053 | 0.251 | 0.050 |
| 79 | 80 | 0 | 105 | 1498 | 0.696 | 0.004 | 0.064 | 0 |
| 80 | 80 | 10 | 115 | 1756 | 0.696 | 0.009 | 0.06 | 0.001 |
| 81 | 80 | 20 | 125 | 1909 | 0.682 | 0.013 | 0.079 | 0.002 |
| 82 | 80 | 30 | 135 | 2194 | 0.681 | -0.02 | 0.054 | 0.005 |
| 83 | 80 | 40 | 145 | 2527 | 0.682 | -0.021 | 0.105 | 0.007 |
| 84 | 80 | 50 | 155 | 2707 | 0.673 | -0.025 | 0.12 | 0.011 |
| 85 | 80 | 60 | 165 | 3029 | 0.666 | -0.034 | 0.155 | 0.02 |
| 86 | 80 | 70 | 175 | 3331 | 0.668 | -0.033 | 0.172 | 0.024 |
| 87 | 80 | 80 | 185 | 3477 | 0.661 | -0.05 | 0.227 | 0.042 |
| 88 | 80 | 90 | 195 | 3714 | 0.657 | -0.063 | 0.28 | 0.064 |
| 89 | 80 | 100 | 205 | 4133 | 0.661 | -0.059 | 0.241 | 0.051 |
| 90 | 90 | 0 | 115 | 1770 | 0.695 | 0 | -0.001 | 0 |
| 91 | 90 | 10 | 125 | 1994 | 0.692 | -0.01 | 0.026 | 0.001 |
| 92 | 90 | 20 | 135 | 2139 | 0.678 | -0.009 | 0.021 | 0.001 |
| 93 | 90 | 30 | 145 | 2475 | 0.677 | -0.047 | 0.162 | 0.025 |
| 94 | 90 | 40 | 155 | 2867 | 0.679 | -0.075 | 0.277 | 0.067 |
| 95 | 90 | 50 | 165 | 3011 | 0.67 | -0.076 | 0.277 | 0.068 |
| 96 | 90 | 60 | 175 | 3307 | 0.664 | -0.068 | 0.27 | 0.062 |
| 97 | 90 | 70 | 185 | 3657 | 0.664 | -0.068 | 0.306 | 0.076 |
| 98 | 90 | 80 | 195 | 3798 | 0.657 | -0.108 | 0.369 | 0.147 |
| 99 | 90 | 90 | 205 | 4010 | 0.65 | -0.118 | 0.399 | 0.172 |
| 100 | 90 | 100 | 215 | 4260 | 0.654 | -0.139 | 0.475 | 0.239 |
| 101 | 100 | 0 | 125 | 2084 | 0.694 | -0.022 | 0.077 | 0.006 |
| 102 | 100 | 10 | 135 | 2335 | 0.691 | -0.048 | 0.173 | 0.028 |
| 103 | 100 | 20 | 145 | 2530 | 0.679 | -0.057 | 0.187 | 0.036 |
| 104 | 100 | 30 | 155 | 2881 | 0.674 | -0.067 | 0.231 | 0.05 |
| 105 | 100 | 40 | 165 | 3148 | 0.673 | -0.072 | 0.299 | 0.071 |
| 106 | 100 | 50 | 175 | 3616 | 0.67 | -0.05 | 0.222 | 0.033 |
| 107 | 100 | 60 | 185 | 3855 | 0.663 | -0.05 | 0.221 | 0.033 |
| 108 | 100 | 70 | 195 | 4199 | 0.659 | -0.045 | 0.219 | 0.03 |
| 109 | 100 | 80 | 205 | 4597 | 0.659 | -0.042 | 0.219 | 0.027 |
| 110 | 100 | 90 | 215 | 4876 | 0.654 | -0.041 | 0.196 | 0.022 |
| 111 | 100 | 100 | 225 | 5073 | 0.65 | -0.081 | 0.325 | 0.099 |

**Supplementary table 2:** The sequence of primers used in this study

| **Primers** | **Sequence** | **Reference** |
| --- | --- | --- |
| Caspase-3 | F: 5′-TTCAGAGGGGATCGTTGTAGAAGTC-3′  R: 5′-CAAGCTTGTCGGCATACTGTTTCAG-3′ | (1) |
| Caspase-8 | F: 5′-GATCAAGCCCCACGATGAC-3′  R: 5′-CCTGTCCATCAGTGCCATAG-3′ | (1) |
| Caspase-9 | F: 5′-CATTTCATGGTGGAGGTGAAG-3′  R: 5′-GGGAACTGCAGGTGGCTG-3′ | (1) |
| FAS | F: 5′-TGAAGGACATGGCTTAGAAGTG-3′  R: 5′-GGTGCAAGGGTCACAGTGTT-3′ | (2) |
| BAX | F: 5′-CGAGAGGTCTTTTTCCGAGTG-3′  R: 5′-GTGGGCGTCCCAAAGTAGG-3′ | (1) |
| ATM | F: 5′-ATAGATTGTGTAGGTTCCGATGG-3′  R: 5′-CATCTTGTCTCAGGTCATCACG-5′ | (3) |
| P53 | F: 5'-CCCAGCCAAAGAAGAAACCA-3'  R: 5'-TCTGAGTCAGGCCCTTCTGT-3' | (1) |
| GAPDH | F: 5′-GCACCGTCAAGGCTGAGAA-3′  R: 5′-AGGTCCACCACTGACACGTTG-3′ | (1) |
| miR-181b-5p | F: 5′-AACATTCATTGCTGTCGGTGGGT-3′ |  |
| U6 | F: 5′-CTCGCTTCGGCAGCACA-3′ | (4) |

**Reference**

1. Chatupheeraphat C, Nantasenamat C, Deesrisak K, Roytrakul S, Anurathapan U, Tanyong D. Bioinformatics and experimental studies of anti-leukemic activity from 6-gingerol demonstrate its role in p53 mediated apoptosis pathway. Excli j. 2020;19:582-95.

2. Das H, Koizumi T, Sugimoto T, Chakraborty S, Ichimura T, Hasegawa K, et al. Quantitation of Fas and Fas ligand gene expression in human ovarian, cervical and endometrial carcinomas using real-time quantitative RT-PCR. British Journal of Cancer. 2000;82(10):1682-8.

3. Lee KW, Tsai YS, Chiang FY, Huang JL, Ho KY, Yang YH, et al. Lower ataxia telangiectasia mutated (ATM) mRNA expression is correlated with poor outcome of laryngeal and pharyngeal cancer patients. Annals of Oncology. 2011;22(5):1088-93.

4. Yan X, Gao M, Zhang P, Ouyang G, Mu Q, Xu K. MiR-181a functions as an oncogene by regulating CCND1 in multiple myeloma. Oncol Lett. 2020;20(1):758-64.

**Supplementary 3:** full-length blot images and data from Figure 2E


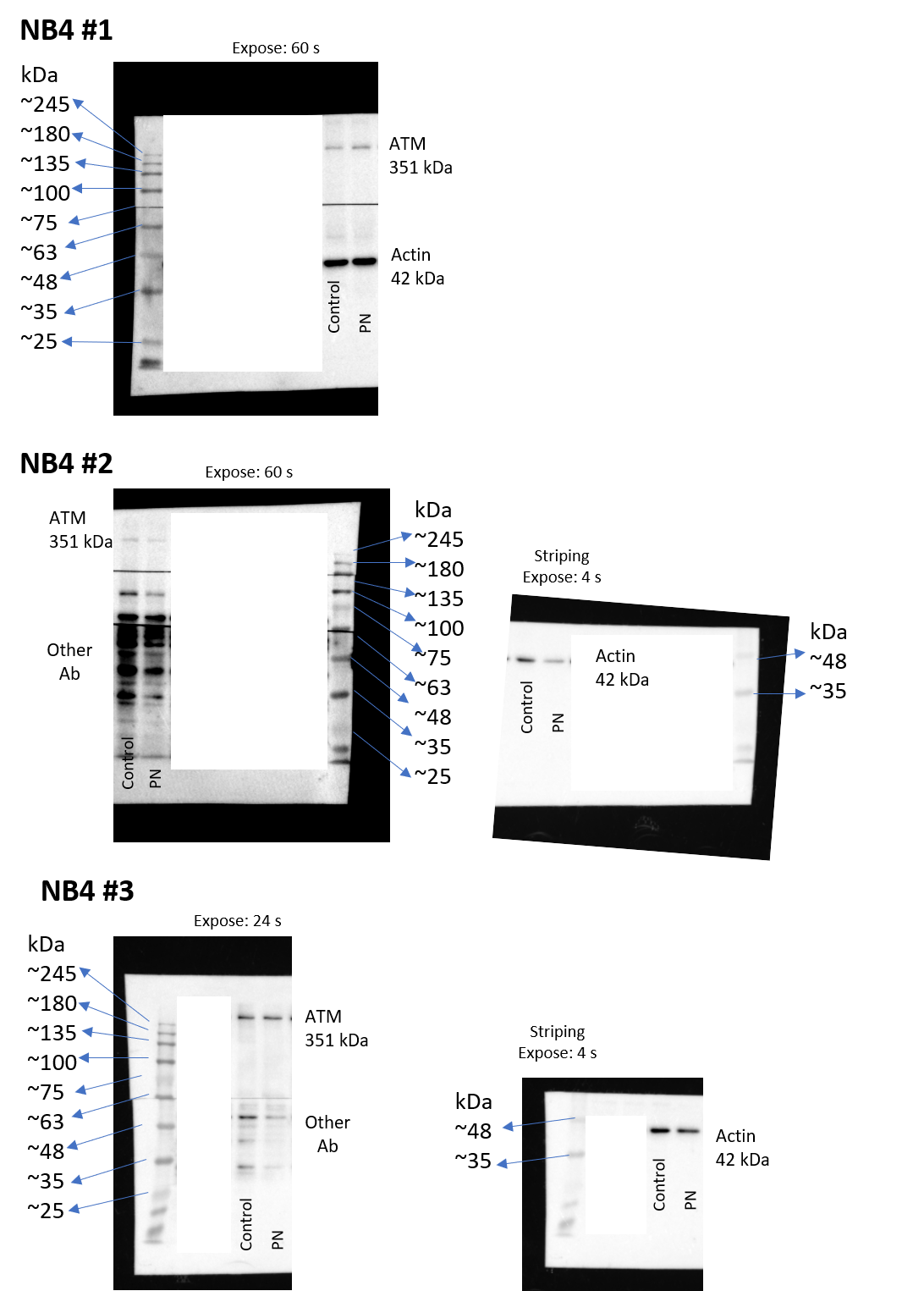


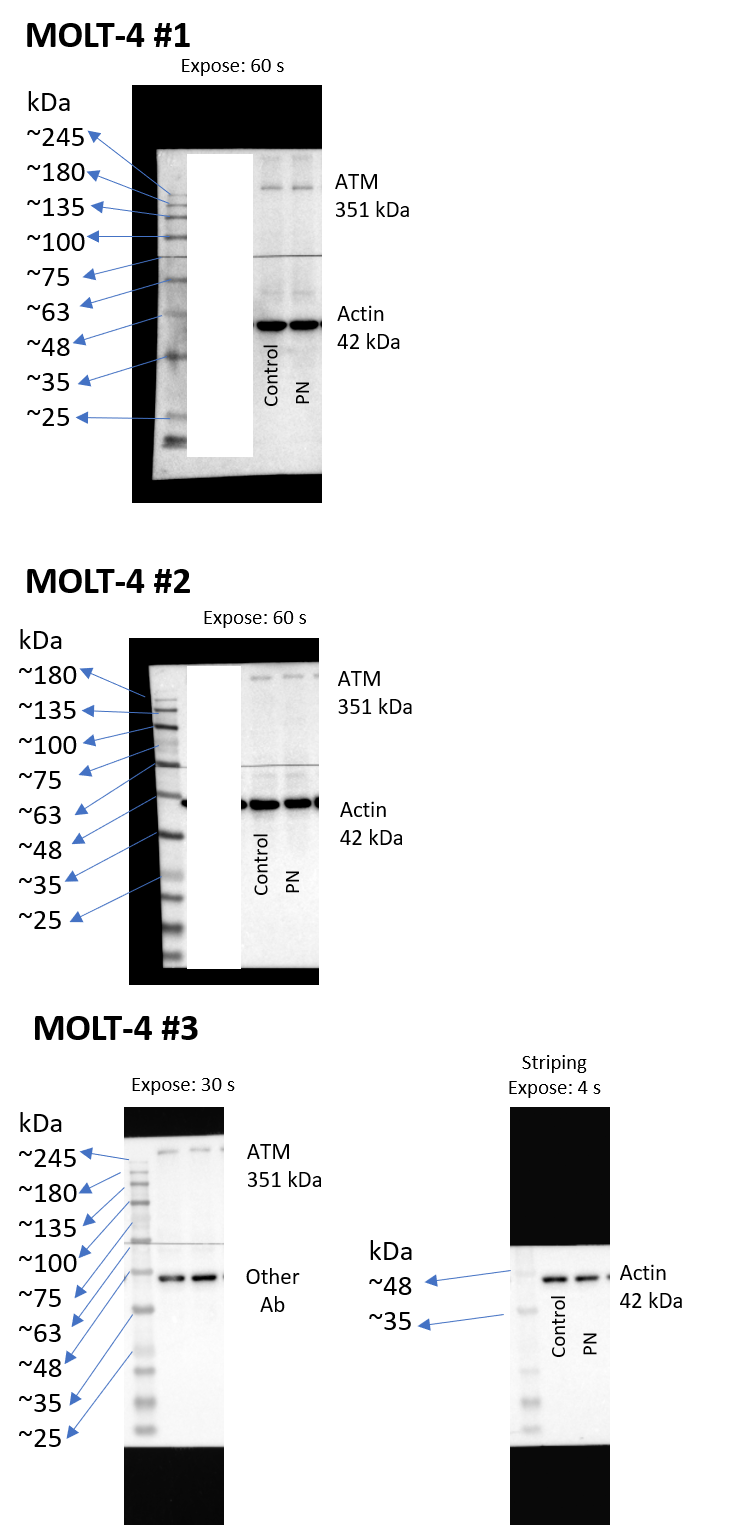


| **Cell** | | | **Band** | **Volume (Int)** | **Normalize** | **Fold change** | **Mean ± SEM** |
| --- | --- | --- | --- | --- | --- | --- | --- |
| **NB4** | I | Control | ATM | 304722 | 0.0831 | 1.41 | 1.34 ± 0.05 |
|  |  |  | Actin | 3666762 |  |  |  |
|  |  | PN | ATM | 458876 | 0.1174 |  |  |
|  |  |  | Actin | 3909378 |  |  |  |
|  | II | Control | ATM | 149226 | 0.0814 | 1.25 |  |
|  |  |  | Actin | 1833027 |  |  |  |
|  |  | PN | ATM | 79306 | 0.1017 |  |  |
|  |  |  | Actin | 779751 |  |  |  |
|  | III | Control | ATM | 1360458 | 0.3983 | 1.35 |  |
|  |  |  | Actin | 3415638 |  |  |  |
|  |  | PN | ATM | 1405206 | 0.5360 |  |  |
|  |  |  | Actin | 2621655 |  |  |  |
| **MOLT-4** | I | Control | ATM | 314204 | 0.0688 | 1.53 | 1.45 ± 0.05 |
|  |  |  | Actin | 4565308 |  |  |  |
|  |  | PN | ATM | 397232 | 0.1056 |  |  |
|  |  |  | Actin | 3762704 |  |  |  |
|  | II | Control | ATM | 197688 | 0.0440 | 1.24 |  |
|  |  |  | Actin | 4491552 |  |  |  |
|  |  | PN | ATM | 188952 | 0.0547 |  |  |
|  |  |  | Actin | 3453696 |  |  |  |
|  | III | Control | ATM | 424515 | 0.1283 | 1.57 |  |
|  |  |  | Actin | 3309856 |  |  |  |
|  |  | PN | ATM | 558684 | 0.2007 |  |  |
|  |  |  | Actin | 2783330 |  |  |  |

**Supplementary 4:** full-length blot images from Figure 4C


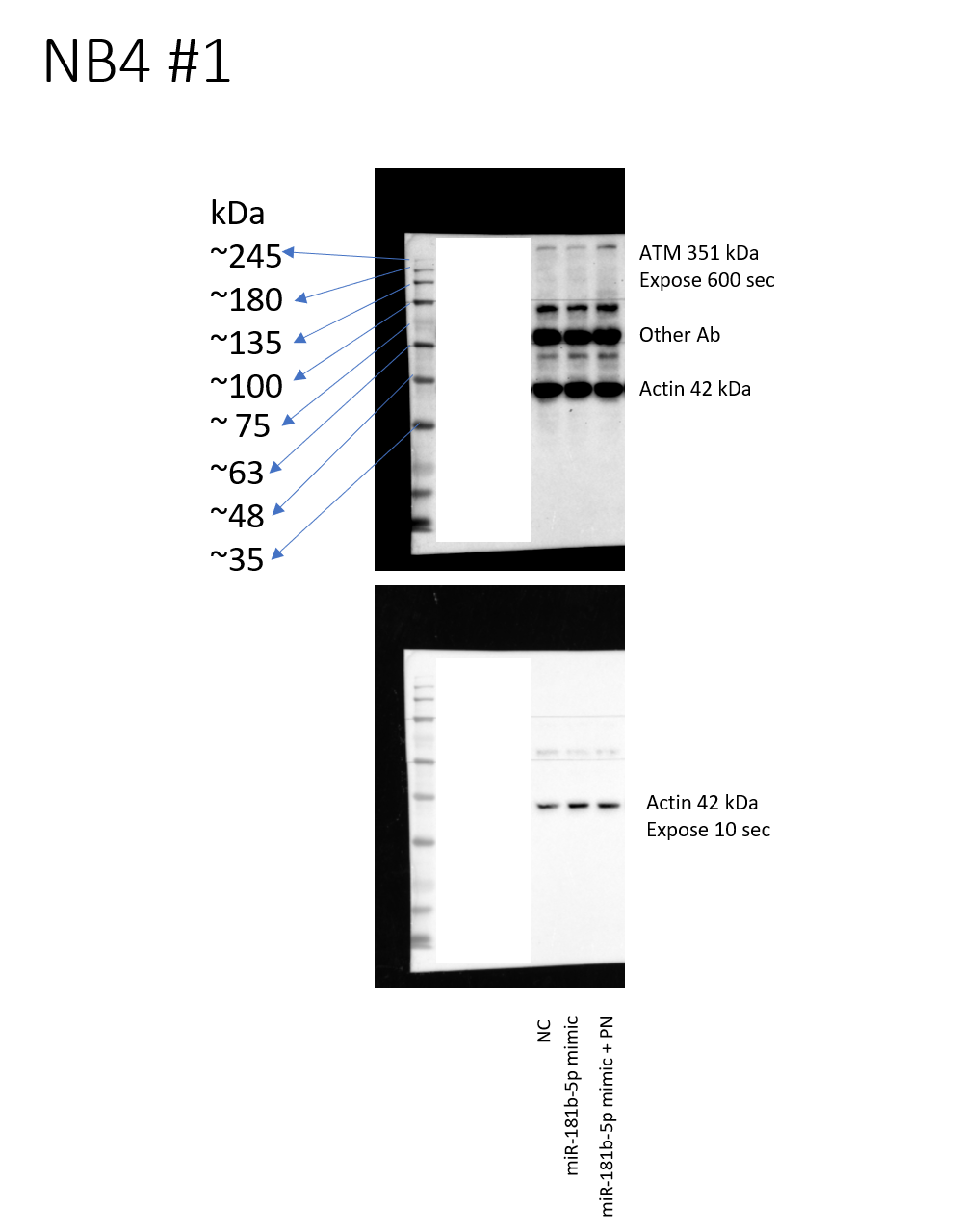


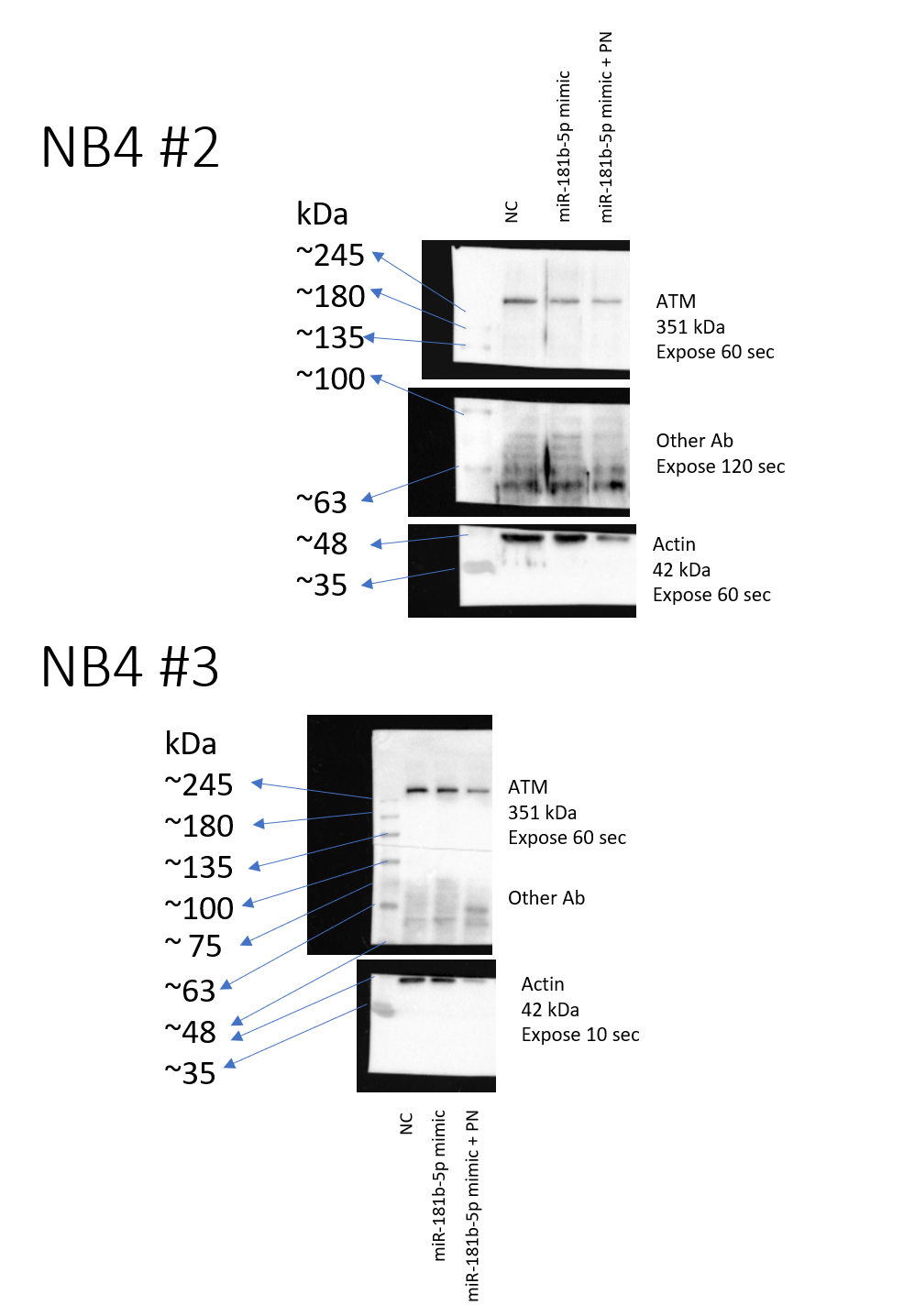


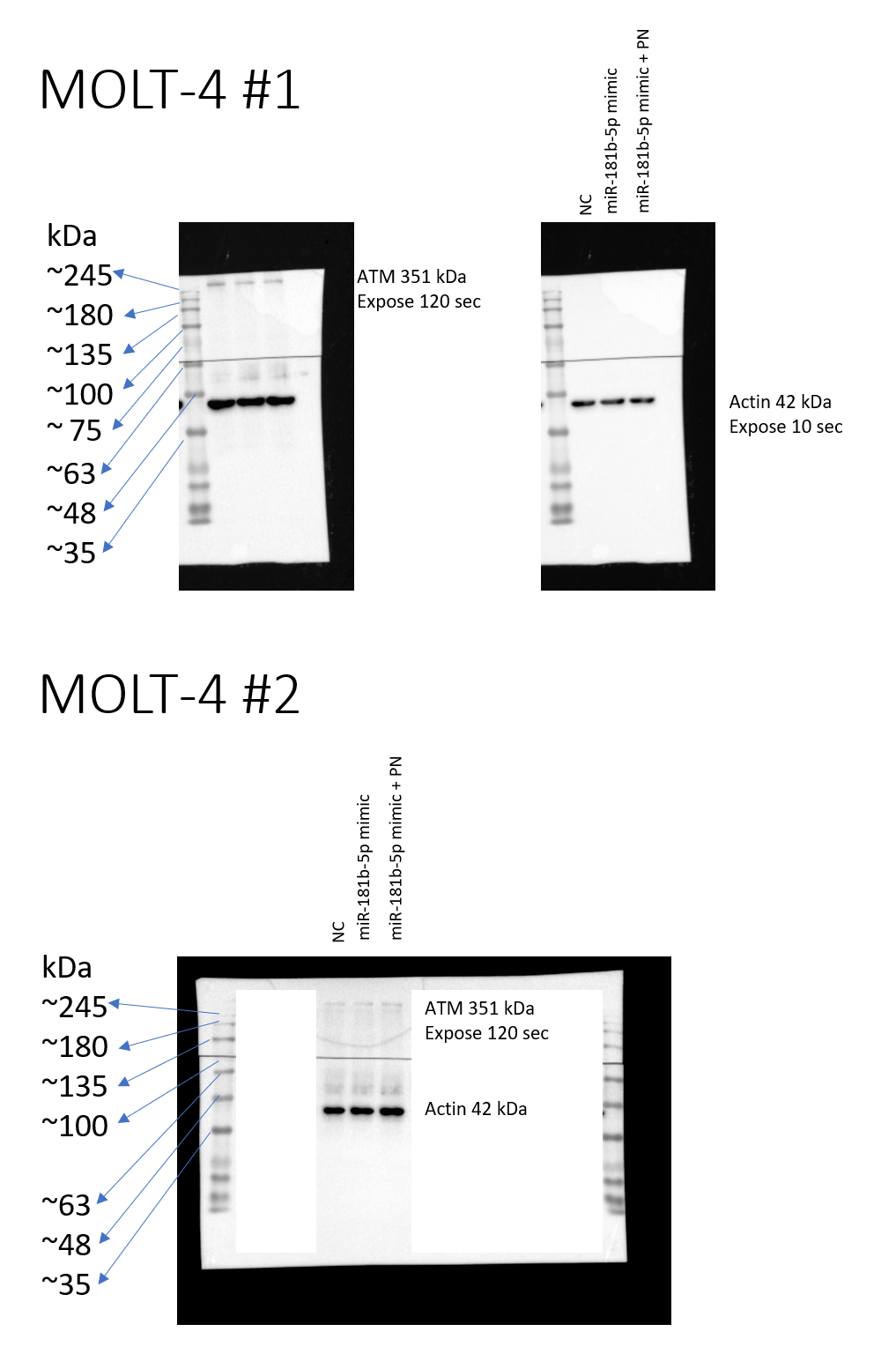


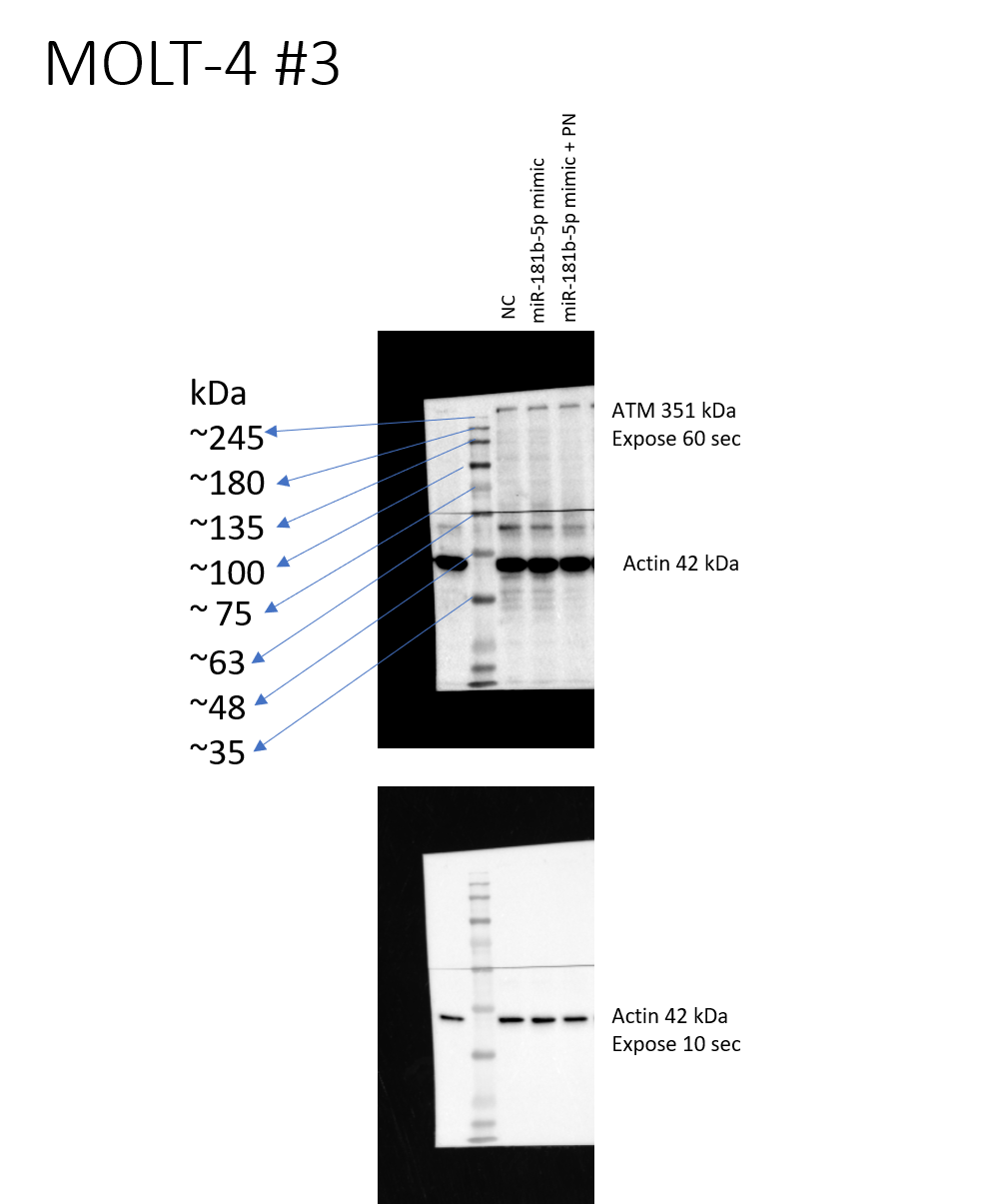

Supplement: Supplementary file 1 — Supplementary Information. [file 41598_2023_35193_MOESM1_ESM.docx]
